# Pancytopenia associated with clonazepam

**DOI:** 10.1186/1756-8722-3-24

**Published:** 2010-07-14

**Authors:** Marnelli A Bautista-Quach, Yu-Min Liao, Chung-Tsen Hsueh

**Affiliations:** 1 Department of Pathology and Laboratory Medicine, Loma Linda University Medical Center, Loma Linda, CA 92354, USA; 2 Department of Internal Medicine, China Medical University Hospital, Taichung, Taiwan, China; 3 Division of Medical Oncology and Hematology, Loma Linda University Medical Center, Loma Linda, CA 92354, USA

## Abstract

We report a case of a 48-year-old Chinese female with end-stage renal disease and chronic anemia on hemodialysis. Clonazepam was prescribed for myoclonus disorder two weeks prior to her hospitalization. Subsequently, she was hospitalized for neutropenic fever with thrombocytopenia and worsening anemia. Bone marrow examination demonstrated a markedly hypocellular marrow (10-20% total cellularity). Clonazepam was discontinued, with gradual improvement of thrombocytopenia, and neutropenia in 1-2 weeks. To our knowledge, this is the first reported case of pancytopenia associated with clonazepam. We recommend patients taking clonazepam to be monitored with regular complete blood count to check for clinically significant pancytopenia or thrombocytopenia.

## Introduction

Clonazepam, a benzodiazepine derivative is used for the treatment of epilepsy, psychiatric, and neurologic disorders [1]. Clonazepam has also been utilized in alleviating movement disorders and restless leg syndrome in patients with end-stage renal disease [2,3]. Cases of thrombocytopenia from clonazepam and other benzodiazepines have been described [4-6]. We report an event of pancytopenia associated with clonazepam in a patient with end-stage renal disease.

## Case Report

A 48-year-old Chinese female with end-stage renal disease on hemodialysis, and mild chronic anemia presented with fever, chills and new-onset leukopenia and thrombocytopenia. She was started on clonazepam (0.25 mg orally twice a day) for myoclonus approximately two weeks prior. Her other medicines included erythropoietin, felodipine, aluminum hydroxide, calcium carbonate, labetalol, folic acid, and daily vitamin B complex. She received two units of packed red blood cells for worsening anemia, with a hemoglobin value of 6 g/dL. Post-transfusion complete blood count (CBC) revealed a hemoglobin of 7.6 g/dL (MCV 92 fL), white blood cell (WBC) count of 460/μL (absolute neutrophil count of 69/μL), and platelet count of 89,640/μL. She subsequently developed fever and chills and was admitted to the hospital the following day.

**Figure 1:**
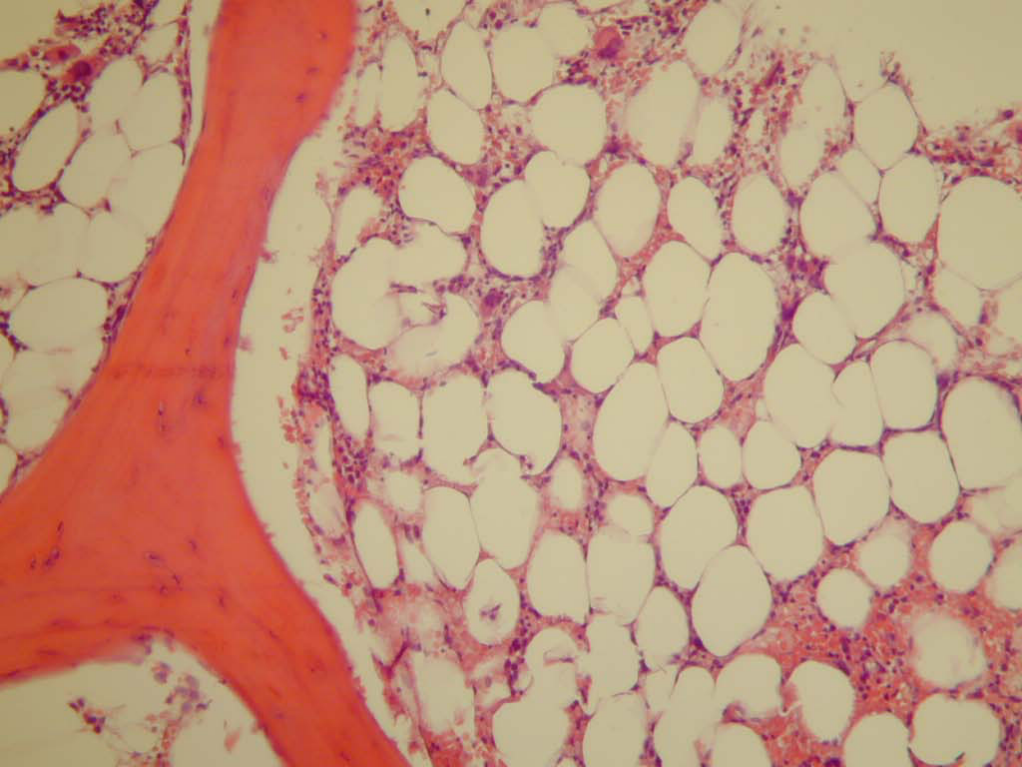
**Bone marrow biopsy (hematoxylin and eosin, 200×)**. Trephine core biopsy showed predominant adipose tissue with significantly decreased hematopoietic elements (10-20% total marrow cellularity).

**Figure 2:**
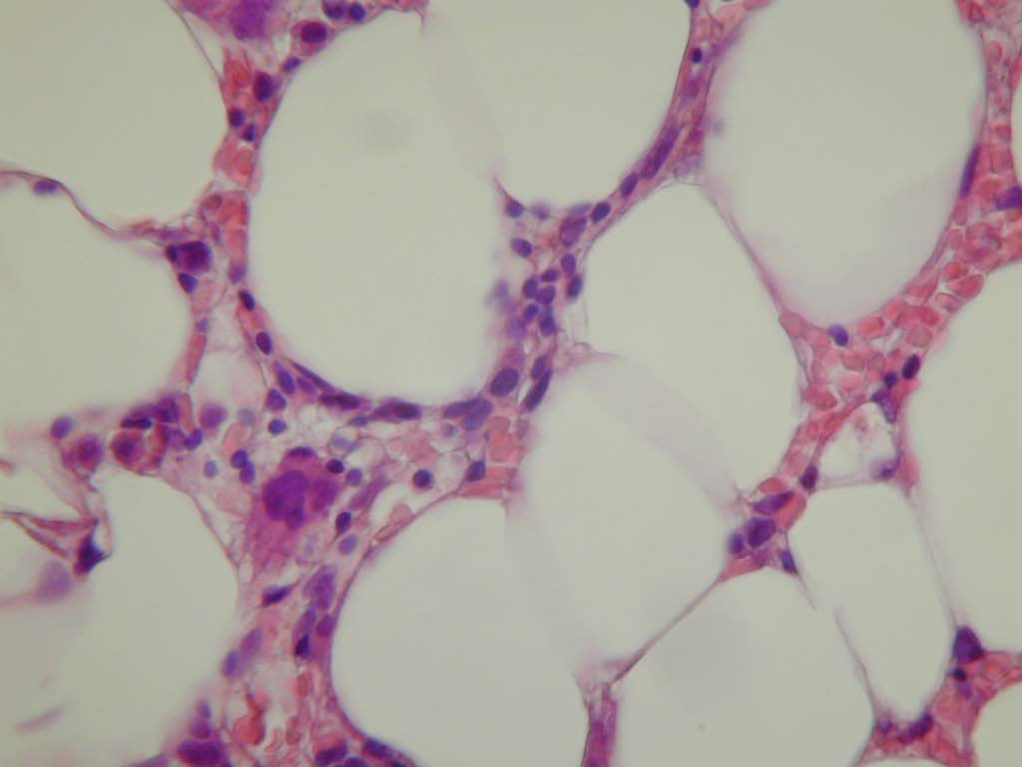
**Bone marrow biopsy (hematoxylin and eosin, 400×)**. Trephine core biopsy showed occasional scattered erythroid and myeloid precursors, and a megakaryocyte.

On the day of admission, CBC showed a WBC count of 386/μL (absolute neutrophil count of 49/μL), hemoglobin of 8.17 g/dL (MCV 91.2 fL), and platelet count of 62,300/μL. Blood culture was obtained which exhibited no growth of microorganisms. She was empirically treated with broad-spectrum antibiotics. Evaluation for human immunodeficiency virus, hepatitis B and hepatitis C viruses were negative. Antinuclear antibody study was non-reactive. Both folate and vitamin B12 levels were within normal ranges. Peripheral blood smear revealed pancytopenia without leukemic blasts. A bone marrow biopsy predominately consisted of adipose tissue, with significantly decreased myeloid and erythroid precursors, as well as megakaryocytes, reflecting a 10-20% overall cellularity (Figs. 1 and 2). No aggregates of blasts or atypical cells were identified. Review of medications suggested that clonazepam, which was added to the patient's regimen two weeks prior to admission, most likely precipitated pancytopenia. Clonazepam was consequently discontinued. Her other medications were maintained. The thrombocytopenia resolved in four days, and neutropenia gradually improved within 1-2 weeks. She was discharged about a week from the day of admission, with CBC showing WBC count of 1,460/μL, hemoglobin of 7.56 g/dL, and platelet count of 246,000/μL.

## Discussion

Several hypotheses have been illustrated in drug-induced aplastic anemia including direct toxic effect to hematopoietic elements, and immune-mediated destruction secondary to idiosyncratic reaction to a drug [7]. El-Sayed and Symonds reported a case of mild pancytopenia in a patient receiving lorazepam and pelvic radiotherapy with a nadir WBC of 2,300/μL, hemoglobin of 11.2 g/dL, and platelet of 90,000/μL [8]. However, bone marrow evaluation was not performed to exclude primary or radiation-related hematologic conditions. Hence, direct effect from radiotherapy could not be completely excluded. Additionally, benzodiazepine-induced thrombocytopenia has been shown to be mediated by platelet-specific antibodies [6].

To our knowledge, our case represents the first reported occurrence of pancytopenia associated with clonazepam. Patients taking clonazepam must be monitored with regular CBC analyses to check for likely development of clinically significant pancytopenia or thrombocytopenia. If indicated, serology may also be pursued to determine the presence of benzodiazepine-dependent antibodies against platelet. Cessation of the drug usually results in gradual improvement of blood counts. Patients should not be re-challenged once hematologic dyscrasia has been documented.

## Consent

Written informed consent was obtained from the patient for publication of this case report and accompanying images. A copy of the written consent is available for review by the Editor-in-Chief of this journal.

## Competing interests

The authors declare that they have no competing interests.

## Authors' contributions

MBQ, YML and CTH performed literature review, and participated in the composition of this case report. YML and CH obtained patient's consent, pertinent clinical data, and photomicrographs. All authors read and approved the final manuscript.
